# Pregabalin versus gabapentin in the treatment of sciatica: study protocol for a randomised, double-blind, cross-over trial (PAGPROS)

**DOI:** 10.1186/s13063-017-2400-y

**Published:** 2018-01-09

**Authors:** Kelvin Robertson, Laurence A. G. Marshman, Maria Hennessy, Linton Harriss, David Plummer

**Affiliations:** 1 Department of Pharmacy, Medical Services Group, The Townsville Hospital, Douglas, Townsville, 4810 Australia; 2 School of Medicine and Dentistry, James Cook University, Douglas, Townsville, 4810 Australia; 3 Department of Neurosurgery, Institute of Surgery, The Townsville Hospital, Douglas, IMB 20, PO Box 670, Townsville, 4810 Australia; 4 Psychology, College of Healthcare Sciences, James Cook University, Douglas, Townsville, 4810 Australia; 5 Centre for Chronic Disease Prevention, College of Public Health, Medical and Veterinary Sciences, James Cook University, PO Box 6811, Cairns, 4870 Australia; 6 Anton Breinl Centre for Public Health and Tropical Medicine, Angus Smith Drive, Townsville, 4814 Australia

**Keywords:** Gabapentin, Pregabalin, Sciatica, Pain, Clinical trial, Protocol

## Abstract

**Background:**

There is currently an absence of high-grade evidence regarding the treatment of chronic sciatica (CS). Whilst gabapentin (GBP) and pregabalin (PGB) are both currently used to treat CS, equipoise exists regarding their individual use. In particular, no head-to-head study of GBP and PGB in CS exists. Despite equipoise, most countries’ formulary regulatory authorities typically favour one drug for subsidy over the other. This hinders interchange wherever the favoured drug is either ineffective or not tolerated. The primary aim of this study is to conduct a head-to-head comparison of the efficacy of PGB versus GBP for CS based on outcomes on a visual analogue scale (VAS) and the Oswestry Disability Index (ODI).

**Methods/design:**

We are conducting a prospective, randomised, double-blind, double-dummy cross-over study. Included patients will be over 18 years old and have unilateral CS with radiological confirmation of corresponding neural compression/irritation. Pregnant women, those with major organ disease, or those with creatinine clearance < 60 ml/minute will be excluded. Patients will continue their current pain medication at study onset, conditional upon dosage consistency during the prior 30 days. Each drug will be titrated up to a target dose (GBP 400–800 mg three times daily, PGB 150–300 mg twice daily) and taken for 8 weeks. The first drug will then be ceased; however, cross-over will be deferred pending a 1-week washout period. Drug efficacy will be assessed using the VAS and ODI. Results of the Health Locus of Control Scale and side effect frequency/severity will be used to determine psychological functioning. Assuming the hypothesis that PGB will display a superior effect, the sample size required is *n* = 38 with 80% power and a 5% type I error rate. Results will be analysed via intention-to-treat methodology.

**Discussion:**

This study will establish the efficacy of PGB compared with GBP in reducing pain in people with sciatica and lead to greater understanding of the treatment options available.

**Trial registration:**

Australian and New Zealand Clinical Trials Registry, 12613000559718. Registered on 17 May 2013.

**Electronic supplementary material:**

The online version of this article (10.1186/s13063-017-2400-y) contains supplementary material, which is available to authorized users.

## Background

Sciatica or sciatic neuralgia, a common form of lumbosacral radiculopathy, is characterised by low back pain which radiates to the leg and which may be accompanied by sensory loss, motor weakness and/or reflex abnormalities. Sciatica is a symptom defined as well-localised leg pain with a sharp, shooting or burning quality that approximates to the dermatomal distribution of the sciatic nerve down the posterior lateral aspect of the leg [1]. It is often associated with numbness or paraesthesia in the same distribution but typically extends beyond the limits of perceived pain in either a dermatomal or sclerotomal anatomical fashion [2, 3]. The term *sciatica* is used by clinicians in different ways: Some refer to any leg pain referred from the back as sciatica; others prefer to restrict the term to pain originating from the lumbar nerve root. Others believe sciatica is a form of ‘neuropathic’ pain caused by compression or irritation of the roots or nerves that comprise the sciatic nerve [1, 4]. Chronic sciatica (CS) is sciatica which has been present for more than 3 months despite active conservative management, including physical therapy. CS may complicate previous chronic low back pain; however, it may also present purely as an isolated phenomenon [1, 4].

The annual prevalence of sciatica varies widely (1.6–43%) with male predominance [4]. Sciatica accounts for 5% of patients with low back pain presenting to primary care practices and 30% have persistent pain for longer than 12 months. Of these 30% presenting to primary care, 20% are already out of work, and 5–15% require surgery. Over half of patients with sciatica will have pain 4 years post-diagnosis, and the socio-economic cost per country per year is estimated to be $128 million for in-hospital care, $730 million for absenteeism and $708 million for disability [5].

Anti-depressants such as tricyclic anti-depressants (TCAs; e.g., amitriptyline) are widely used to treat neuropathic pain (NP), including CS, as first-line therapy after failure of simple analgesics. On the basis of ‘moderate-quality’ evidence, The National Institute for Health and Care Excellence – United Kingdom (NICE-UK) reported TCA efficacy over placebo for NP [6]; however, on the basis of ‘high-quality’ evidence, TCAs were also significantly more likely than placebo to produce side effects (SEs). Extrapolating NICE-UK guidelines, prescribing authorities (e.g., Australian Therapeutic Guidelines [ATG]) often insist on trialling TCAs first for NP prior to introducing second-line agents. Limited information, however, is available regarding TCA use in CS. In one rare cross-over study, nortriptyline—alone or combined with morphine—had no significant benefit over placebo [6].

Anti-convulsant anti-neuropathic agents such as gabapentin (GBP) and pregabalin (PGB) are also widely used to treat NP, including CS. On the basis of ‘moderate- to high-quality’ evidence, NICE-UK noted the efficacy of these agents over placebo for NP [6]. Australian prescribing authorities (e.g., ATG) recommend anti-neuropathic agents as second-line agents for NP, even though NICE-UK did not actually favour TCAs over anti-neuropathics as first-line agents (or vice versa). However, NICE-UK states that when introducing second-line agents, ‘overlap’ with pre-existent regimens should be considered to avoid decreased pain control [6]. A recent literature review provides information on the individual efficacy of PGB and GBP over placebo for CS; however, when compared head-to-head, no firm conclusions can be made [7].

In summary, sciatica, like most NP states, often proves resistant to simple analgesic regimens (including paracetamol, non-steroidal anti-inflammatory drugs [NSAIDs] or opioids) and recommended first-line TCAs [1, 4]. Instead, the drugs most commonly used currently in both CS and NP are GBP or PGB [1, 4]. PGB and GBP are both analogues of γ-aminobutyric acid, a substance known to modulate calcium channel subunits. Both GBP and PGB may therefore possibly act by decreasing neurotransmitter release associated with central sensitisation in both CS and NP.

As with NP, there is currently an absence of high-grade evidence regarding the medical treatment of CS [1, 6]. No adequately powered direct ‘head-to-head’ trials comparing either PGB or GBP with other drugs are extant [1, 6]. Indirect comparisons, using placebo as the common comparator, have been published; however, each has represented differing patient populations, differing primary outcomes and differing pain measurement scales [6]. Authors of a recent review concluded, albeit based on weak evidence, that efficacy and SEs with GBP and PGB were probably similar [7].

Notwithstanding this information, citing minor titration but definite cost advantages, NICE-UK nevertheless favoured PGB over GBP [6]. However, costs of either PGB or GBP vary widely globally. Moreover, costs vary unpredictably (i.e., PGB more expensive than GBP or vice versa) on a global basis [7]. Despite this, formulary regulatory authorities in most countries have, like NICE-UK, favoured one drug over the other. Furthermore, and somewhat paradoxically, formulary regulatory authorities in most countries have typically favoured the more expensive drug, whether GBP or PGB [7]. For example, GBP is currently available on the Australian Pharmaceutical Benefits Scheme (PBS) in Australia and some hospitals in the United Kingdom only for epilepsy; it is not listed for NP. PGB, by contrast, is subsidised on the PBS for NP. The U.S. Food and Drug Administration, along with Health Canada, have adopted reimbursement criteria similar to that of the Australian PBS; notwithstanding this, both GBP and PGB can be accessed in the United States and Canada via special access schemes (if patients satisfy stringent criteria for NP). In marked contrast, GBP is listed for use in treating both partial seizures and NP throughout Europe. The rulings of formulary regulators have therefore been inconsistent and dependent upon the individual body. Such action hinders interchange wherever the favoured drug is either ineffective or not tolerated [7]. Given that no evidence supports unhindered PGB-GBP interchange and that no study has directly challenged GBP and PGB head-to-head, neither GBP nor PGB should probably be favoured, given current evidence [7].

Prospective ‘head-to-head’ studies are therefore urgently required to provide a robust evidence base for GBP or PGB use in sciatica [4]. Both medications have previously displayed efficacy when compared with placebo [1, 8, 9]. We therefore aimed to perform the first study to assess GBP and PGB directly head-to-head for treatment of CS.

### Objectives

#### Primary objective and outcome

Our primary objective is to demonstrate if either GBP or PGB demonstrates superiority over the other in terms of efficacy for the treatment of patients diagnosed with CS. The co-primary outcome is leg pain intensity using a visual analogue scale (VAS) measured at baseline and at weeks 4, 8, 10, 14 and 18. The participants will be asked to rate their average leg pain over the last 24 h on a scale of 10, with zero representing ‘no leg pain’ and 10 representing the ‘worst pain imaginable’ [10].

The co-primary outcome is the Oswestry Disability Index (ODI) [10], measured at baseline and at weeks 4, 8, 10, 14 and 18, to assess disability. The Health Locus of Control Scale (HLOC) will also be used at baseline and at weeks 4, 8, 10, 14 and 18 to assess participants’ decision-making processes because we have identified compliance with these medications as being low [10].

#### Secondary objective and outcome

Our secondary objective is to demonstrate if one drug (i.e., either GBP or PGB) demonstrates superiority over the other in terms of the frequency and severity of SEs in the treatment of sciatica. The key secondary outcome will be the record of frequency and severity of SEs. Details of SEs will be collected at weeks 4, 8, 10, 14 and 18. The most common SEs of PGB are dizziness and somnolence [11]. The most common SEs of these medications are dizziness (27%), drowsiness (22%) and decreased memory (20%) [10].

## Methods/design

**Fig. 1:**
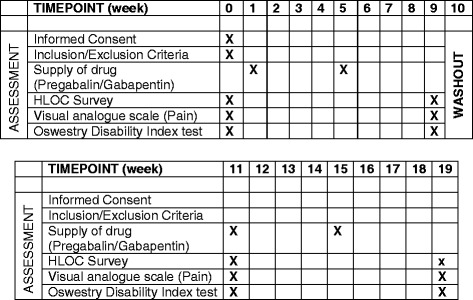
Standard Protocol Items: Recommendations for Interventional Trials (SPIRIT) figure for Pregabalin and Gabapentin Prospective Clinical Trial for the Treatment of Sciatica: A Randomised, Double-Blind, Cross-over Study (PAGPROS). *HLOC* Health Locus of Control Scale

**Fig. 2:**
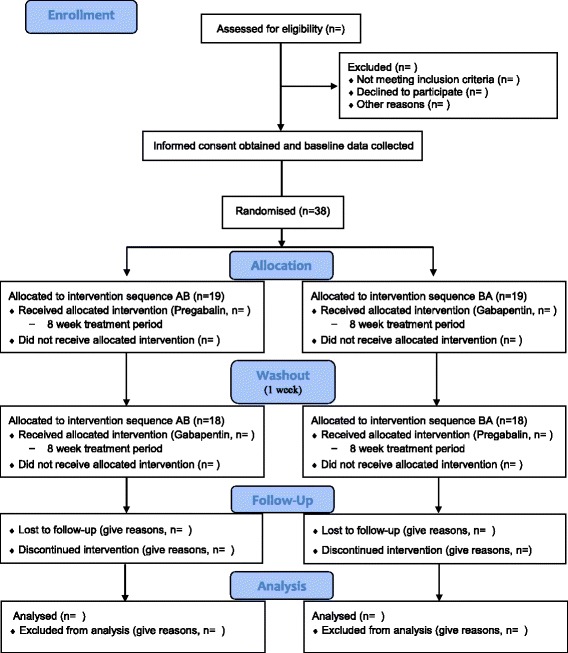
Consolidated Standards of Reporting Trials (CONSORT) flow diagram for Pregabalin and Gabapentin Prospective Clinical Trial for the Treatment of Sciatica: A Randomised, Double-Blind, Cross-over Study (PAGPROS)

The Pregabalin and Gabapentin Prospective Clinical Trial for the Treatment of Sciatica: A Randomised, Double-Blind, Cross-over Study (PAGPROS) is a double-blind, randomised, double-dummy, cross-over trial comparing PGB with GBP for the treatment of CS (Fig. 1). Ethics approval was obtained from the local human research ethics committee, and the study has been registered with the Australian and New Zealand Clinical Trials Registry (ANZCTR, 12613000559718). The study protocol follows the Standard Protocol Items: Recommendations for Interventional Trials (SPIRIT) statement [12] (Additional file 1), and a SPIRIT figure and CONSORT diagram are provided in Figs. 1 and 2.

### Participants and recruitment

Participants with unilateral CS will be recruited from attendance at a specialist neurosurgery clinic in a large tertiary hospital located in Townsville, Australia. The study specialists, comprising consultant neurosurgeons, will perform a medical evaluation to gain relevant medical and medication history and screen the patient against the eligibility criteria. This initial intervention will include baseline scores for VAS, ODI and HLOC. The patient will then be directed to the trial pharmacist, who will remain independent of the treating team, for consent and signature of the informed consent document.

Patients are deemed eligible if they meet all of the following criteria:

- Pain radiating into one leg only to, at or below knee level
- Magnetic resonance imaging/computed tomography-confirmed sciatica caused by a degenerative condition (e.g., degenerative disc disease, bone spur growth, degenerative scoliosis)
- Naive to PGB and GBP use
- Aged 18 years or older
- Sufficient understanding of the English language or interpretation assistance available to complete the study treatment and assessments

Concomitant medication, including analgesics and central nervous system CNS depressants (paracetamol, NSAIDs, and opioids), can be continued as long as the medication dose has been stable for 30 days prior to the start of the study.

Patients will be excluded if they meet any of the following criteria:

- Pregnant or breastfeeding women or females planning conception during the study period
- Patient history or laboratory results that suggest the presence of inherited neuropathy or neuropathy attributable to other causes (hypothyroidism, vitamin B_12_ deficiency, connective tissue disease, amyloidosis, toxic exposure)
- Major organ system disease, diabetic cardiovascular autonomic neuropathy with abnormality in sympathovagal balance, baseline postural hypotension of more than 20 mmHg
- Specific contraindications to PGB or GBP (allergy to or significant renal impairment); PGB and GBP are both predominantly renally excreted, so patients with an estimated creatinine clearance < 60 ml/minute will be excluded
- Other neurologic medications such as serotonin reuptake inhibitors (paroxetine, fluoxetine), dual (serotonin and noradrenaline) reuptake inhibitors (venlafaxine), benzodiazepines, anti-convulsant medications (valproic acid, carbamazepine), anti-psychotic medications (clozapine, olanzapine, risperidone) or bipolar disorder medications (lithium)
- People with a diagnosis of cancer, dementia, severe mental illness or other condition which will significantly reduce the ability to consent and/or fully undertake the program
- Diabetic and/or HIV-related neuropathies

If a patient is eligible, the unblinded independent trial pharmacist will gain informed consent and notify the research team. The participant will then be randomised, and the pharmacist will dispense and counsel on the study medications and arrange visit appointments with reminders. At this point, baseline data will be confirmed by the pharmacist as collected at the first visit, or subsequently via telephone, before the participant commences the study medication. Following baseline data collection, the researcher will instruct the participant to break the seal on the medication pack and commence the study medicine as per the dosage instructions. At this point, the participant is considered to have been included in the study.

To ensure consistency, the study researchers will ensure that the protocol is being followed and that good clinical practice is being monitored. General practitioners will be able to refer community patients into the trial via a trial specialist hotline contact number whereby the patient is screened by a study specialist to ensure consistency of enrolment.

### Randomisation and blinding

The trial pharmacist (un-blinded) will generate a randomisation code using a computer-derived permuted block with varying block size sequence. Manufacturing and preparation of the medication capsules will be performed by an external good manufacturing practice-accredited facility. The unblinded pharmacist will be involved in the preparation of the medication kits as per the randomisation schedule. The sequence will follow a 2 × 2 sequential design whereby participants will receive PGB first, then GBP (or vice versa), in a double-blinded fashion. Owing to the variability in regular dosage frequency between the medications (PGB twice daily, GBP thrice daily), study medication packs will contain three bottles each, correlating to the dosage times of morning, lunchtime and night, so as to maintain blinding. Medication packs pertaining to the PGB arm will have placebo incorporated as the lunchtime dose with all medications being indistinguishable. The randomisation schedule will remain concealed from other researchers. Placebo capsules will have an appearance identical to the active capsules. The randomisation process will ensure concealed allocation and blinding of the specialist, the participant and the outcome assessor.

### Study treatment

Participants will be randomised to commence treatment on either PGB or GBP. As a result of the cross-over methodology, participants will have the opportunity to experience both PGB and GBP, and we predict little or no carry-over effects (medium- or long-term) after the washout period. We believe the incorporation of a stand-alone placebo arm is unethical in trials where participants with moderate to severe pain are recruited.

The starting dose of PGB is 150 mg once daily for the first week. This will be titrated to the participant’s optimal dose up to a maximum of 300 mg twice daily, depending on their progress and tolerance at each dose level. The starting dose for GBP is 400 mg once daily for the first week. This will be titrated to the participant’s optimal dose up to a maximum of 800 mg thrice daily, depending on their progress and tolerance at each dose level. These doses are based on national recommendations from the Australian Medicines Handbook [11]. In the standard study dosing regimen (Table 1), we expect a 4-week titration period, after which the maximum tolerated dose for each participant will be maintained for 4 weeks before the first study medication is ceased in preparing for washout. The washout period between treatment phases will last 1 week, which is sufficient for these medications because they possess a short half-life (5–7 h). The dosage of either PGB or GBP can be amended at any stage in PAGPROS on the basis of efficacy and/or SEs by communication between the study specialist and the study pharmacist. The maximum treatment period is 8 weeks [13].

**Table 1:** PAGPROS medication titration schedule

	Medication	Total daily dose
Week	Pregabalin	
1	1 × 150-mg capsule in the morning	150 mg/day
2	1 × 150-mg capsule three times daily (middle dose is placebo)	300 mg /day
3–8	2 × 150-mg capsules three times per day (middle dose is placebo)	600 mg/day
Week	Gabapentin	
1	1 × 400-mg capsule in the morning	400 mg/day
2	1 × 400-mg capsule three times per day	1200 mg/day
3–8	2 × 400-mg capsules three times per day	2400 mg/day

*PAGPROS* Pregabalin and Gabapentin Prospective Clinical Trial for the Treatment of Sciatica: A Randomised, Double-Blind, Cross-over Study

The titration and dosage regimen are based on recommendations from clinical practice and medication guidelines such as the Australian Medicines Handbook and product prescribing information. Both medications have the potential for adverse neurological SEs, and hence a slow ascent in dose will contribute to mitigating this risk for participants and increasing compliance with the trial protocol. Simultaneously prior to washout, the dosage will be gradually reduced instead of being abruptly halted, further decreasing the likelihood of medication misadventures for the participants (and increasing compliance).

In addition to PGB or GBP, participants may continue concomitant medications (including analgesics) as long as the dosage has been stable for 30 days prior to commencing the study period. These concomitant medications will be closely monitored and recorded in the case report form (CRF). Medicines for NP include anti-depressants, selective serotonin and noradrenaline reuptake inhibitors, topical lignocaine and other anti-convulsant medications [14]. Note that this practice is entirely consistent with NICE-UK guidelines, which state that when super-adding second-line agents for analgesic control (such as GBP and PGB), ‘overlap with first-line agents is encouraged to avoid decreased pain-control’. To our knowledge, only one prospective cohort study has reflected this practice with GBP in CS [14]. However, participants should not take concomitant medication that could result in an adverse interaction with PGB or GBP, including medicines that might increase the risk of excessive sedation (for example, benzodiazepines) [11]. No other pain interventions will be permitted throughout the study period; if considered necessary, then such patients will be withdrawn from PAGPROS.

Participating in PAGPROS is completely voluntary, and participants can stop taking part at any time without explanation or prejudice. Ceasing to participate in PAGPROS may be considered, for example, wherever participants wish to explore the possibility of other treatments, including other medications or interventions (*see above*). In some cases, participants may find that the starting dose of either PGB or GBP, whilst efficacious, produces unwanted SEs [10]. In such cases, a lower dose may be required, at least for a period of time. Because this cannot be accommodated within the current PAGPROS protocol, such patients will be removed from the study, and their data will be analysed as per intention-to-treat (ITT) principles; however, they may still form part of a prospective cohort for parallel study.

### Data collection

Data collection will be conducted by the study researchers via telephone, email or online at baseline (before medication commencement) and at weeks 4, 8, 10, 14 and 18. Week 10 data collection will act as the cross-over secondary baseline for analysis purposes. Data will be entered into CRFs by dedicated trained staff. Each participant will receive up to seven face-to-face or telephone consultations with the trial pharmacist to commence treatment, monitor progress and adjust the dose of the study medication over the 8-week treatment periods. These visits will also incorporate a medical evaluation and collection of primary and secondary outcomes. Participants will receive usual neurosurgical care independent of and parallel to PAGPROS.

The use of prior and continued analgesic medicines will be collected at baseline. Adherence to study medication will be documented through a self-reported daily medication diary and by counting the returned medicine compared with the prescribed regimen as recorded by the trial pharmacist. Participants will be asked to return used and unused study medications at each visit.

### Data integrity and analysis

The integrity of trial data will be monitored by regularly scrutinising data files for omissions and errors. We will perform double data entry of the primary and key secondary outcomes. The source of any inconsistencies will be explored and resolved. Electronic data will be stored on a secure server, and paper copies will be locked in a cabinet. Data will be accessible only by researchers, and participant confidentiality will be maintained through secure password-protected data storage during and after PAGPROS.

Data will be de-identified prior to statistical analysis, which will be performed on an ITT basis. Normality of data distribution will be assessed, and appropriate parametric (Student’s *t* test or analysis of variance) or non-parametric (Wilcoxon signed-rank test, Wilcoxon rank-sum tests) tests for between-group differences will be performed. Statistical significance will be assessed at *p* < 0.05. Subgroup analysis may be implicated and considered as PAGPROS develops. Time-to-event analysis will be undertaken using Kaplan-Meier estimates on the week 8 and week 18 VAS scores. Missing data will be handled by a single imputation method whereby the last observation will be carried forward and used as a surrogate for the missing value. This method is the favoured approach for replacing missing data because it is conservative, yields an appropriate estimate of variation in outcome and is unlikely to bias towards the alternative hypothesis [15]. An alternative approach to missing data may be use of a longitudinal mixed-effects model incorporated into the analyses.

### Sample size

We hypothesise that over an 8-week treatment period, GBP will reduce pain on the VAS scale by an average of 4.5 points from (7.5 to 3.0) as per historical literature [16]. We predict PGB to show at least the same benefit. We hypothesise that PGB will display superiority over GBP by at least a 20% better relative reduction in VAS score, with a resultant reduction of 5.4 (from 7.5 to 2.1) points from baseline. This 20% relative reduction is based on the average reduction of pain symptoms compared with placebo for indirect comparisons [1, 16]. Relative reduction will be used because it is often more impressive and also to allow for the instance of a lower-than-expected event rate, which would lower the absolute risk reduction.

If the true difference in means of both arms of the study is 0.9 with an SD of 1.2, in order to detect this 20% relative decrease in pain between GBP and PGB, we will need to study 30 patients (15 per treatment arm) to reject the null hypothesis with 80% power. The type I error probability associated with this test of the null is 0.05. Assuming a 20% drop-out rate, the total sample size will be 38 patients (19 per treatment arm). We have chosen this large effect size and conservative SD on the basis of anecdotal and specialists’ experience with this cohort of patients. The benefits of the cross-over methodology are evident with the small sample size required, owing to each participant acting as his/her own control. If this were a conventional parallel study design, the sample size needed would be approximately 100 participants. We conservatively estimate that if two people can be recruited per week, the study duration will be approximately 1.5 years.

### Adverse experiences and monitoring

Potential risks of both PGB and GBP have been well studied owing to their use for neuropathic conditions. These risks have been minimised by our exclusion criteria. Any SEs will be monitored weekly during follow-up phone calls and examinations. Close monitoring of other neurological pain medications will be done with patient diaries. SEs were quantified in a latest meta-analysis and given the rare SEs of both medications and their likely effectiveness, and the potential benefits outweigh the risks in this study [1].

During the recruitment period, a monitoring visit may be applicable. The responsible monitor will be a specialist neurosurgeon who is not involved in the conduct of the trial and is chair of the hospital patient safety committee. The purposes of monitoring are as follows:

- To ensure that the study is conducted according to the protocol and applicable guidelines and regulations
- To verify source data against data on the CRF and in the database
- To check the security of stored data
- To confirm that the consent process, approved by the Townsville Hospital Human Research Ethics Committee, has been followed and to view a random sample of original signed consent forms
- To review all serious adverse events (SAEs)

Interim data monitoring will take place in-house for review of safety and SEs. The trial may be stopped if more harm to patients is shown. The Pocock boundary will be used as the stopping rule, whereby after each set of 2n patient responses to a total of ‘K’ looks at the data. "2n" patient responses means there will be at least 2 interim analyses whereby the stopping rule can be enforced. This will be a group sequential approach whereby the critical boundary (*p* < 0.018) will be set at each look.

An adverse event is the appearance or worsening of any undesirable sign, symptom or medical condition occurring after starting the study, even if the event is not considered to be related to the investigational drug. Any SAE (defined as an event that is life-threatening or results in death, hospitalisation or significant disability) will be reported immediately to the relevant authorities (study monitor, ethics committee, data and safety monitoring board). If a potential relationship is suspected between the study drug and an SAE, then un-blinding to treatment allocation is indicated, and the participant will be withdrawn from PAGPROS.

Abnormal laboratory values or test results constitute adverse events only if they induce clinical signs or symptoms, are considered clinically significant or require therapy. The occurrence of adverse events should be sought by non-directive questioning of the patient at each visit during the study. Adverse events also may be detected when they are volunteered by the patient during or between visits or through physical examinations, laboratory tests or other assessments.

All adverse events will be recorded as follows:

- Severity grade: mild, moderate or severe
- Relationship to investigational drug: suspected/not suspected
- Duration
- Continuation to an SAE

All adverse events will be treated appropriately. The action taken to treat the adverse event should be recorded. An SAE is defined as follows:

- Fatal or life-threatening
- Results in persistent or significant disability/incapacity
- Constitutes a congenital anomaly/birth defect
- Requires inpatient hospitalisation or prolongation of existing hospitalisation

To ensure patient safety, every SAE, regardless of suspected causality, occurring after the patient has provided informed consent and until 7 days after the patient has stopped study participation will be noted by expedited reporting to the Townsville Hospital and Health Service Human Research Ethics Committee.

### Modification of the protocol

Any modifications to the protocol that may impact the design and conduct of the study will require a formal protocol amendment. Such amendment will be agreed upon by the study investigators and approved by the local ethics committee prior to implementation. Once approved, the changes will be communicated to the relevant parties.

## Discussion

The PAGPROS protocol presents the design and rationale for a double-blind, double-dummy, randomised cross-over trial comparing the efficacy of PGB with GBP in treating CS. Owing to the variability in regular dosage frequency between the medications (PGB twice daily, GBP thrice daily), study medication packs will contain three bottles each, correlating to the dosage times of morning, lunchtime and night, to maintain blinding. Medication packs pertaining to the PGB arm will have placebo incorporated as the lunchtime dose, with all medications being indistinguishable.

Thus, PAGPROS represents the first head-to-head study to determine the relative role of either PGB or GBP in the evidence-based medical management of CS. However, in addition to efficacy, PAGPROS will also determine the frequency and severity of SEs with PGB or GBP. Thus, PAGPROS will determine the ‘efficacy versus SE trade-off’ with each drug and whether differences in compliance rates result in consequences. For example, in a prior study with GBP in treating CS, 31% of patients ceased GBP within 1 week of treatment [10]. Moreover, efficacy was significantly less in those who experienced SEs in that study [10].

In PAGPROS, we will employ the HLOC to assess psychological functioning with PGB or GBP in CS. In particular, PAGPROS will explore the prognosis of each drug relating to questionnaire outcomes relating to patients’ insight into their psychological dysfunction. Thus, PAGPROS may determine deficits and provide information not actually reported as SEs by the patients themselves. This may prove to be an important aspect of the study. For example, a prior prospective cohort study with GBP in treating CS revealed that, of 23 different SE types amongst 53% of patients, more than half could have adversely affected the ability to drive a motor vehicle safely or even to maintain employment [10].

Finally, the double-blind cross-over design of PAGPROS may provide guidance regarding the implications of any potential need to substitute one drug for the other. For example, PAGPROS may determine whether SEs experienced with one drug are also observed with the other (i.e., in the same patient, in close temporal succession after cross-over). This may prove especially important should PAGPROS demonstrate a between-groups null effect regarding efficacy. However, PAGPROS may show significant efficacy to one drug but no efficacy to the other. Despite a lack of an evidence base, many formulary regulatory authorities worldwide typically favour one drug for subsidy over the other [4]. This hinders interchange wherever the favoured drug is either ineffective or not tolerated [4]. The nature of PAGPROS’s design will directly assess the utility of cross-over between PGB and GBP and will therefore enable formulary regulatory authorities to make more informed therapeutic decisions than currently.

Recruitment commenced in early 2016, with data collection to be completed by mid 2018. The allocation concealment and double-blind design minimise bias, and data collection processes ensure data quality and integrity. The trial team has extensive experience in the design, conduct and reporting of clinical trials. Results of the study will be disseminated via publications and presentations.

### Potential weaknesses of PAGPROS

#### Treatment duration

PAGPROS permits a 4-week titration period, after which the maximum tolerated dose for each participant will then be maintained for 4 weeks. The duration of individual drug study is therefore 8 weeks. In some rare cases, this might be considered insufficient to test efficacy at the optimum dose [10]. Furthermore, because anecdotally some patients develop tolerance to SEs incurred with either PGB or GBP, the study period may also be too short to detect SE tolerance.

#### Dosages

Given the restricted doses and study time available in PAGPROS, it is not possible to introduce either drug in ‘low and slow’ fashion [4]. Because the latter potentially off-sets the development of SEs [4], PAGPROS may therefore potentially over-estimate SEs with either drug. However, at least with GBP, there exists some control in that a prospective cohort study found SEs in 53% of patients with CS [10].

#### Maintenance of background therapies including prior analgesia

This may affect both efficacy and SE development, potentially increasing both. However, note that this practice is entirely consistent with NICE-UK guidelines [6, 10], and, indeed, standard clinical practice. NICE-UK guidelines state that, when super-adding second-line analgesic agents (such as GBP and PGB), ‘overlap with first-line agents is encouraged to avoid decreased pain-control’ [6]. To our knowledge, only one prospective cohort study has reflected this practice using GBP in CS [10].

### Trial status

Screening for patients for this trial began on 7 March 2016. The first patient was included on 4 April 2016. To date, 18 participants have successfully completed the trial, and inclusion is expected to run until 31 June 2018.

## Additional file

Additional file 1: SPIRIT 2013 checklist: recommended items to address in a clinical trial protocol and related documents. (PDF 131 kb)

## Abbreviations

ATG: Australian Therapeutic Guidelines
CONSORT: Consolidated Standards of Reporting Trials
CRF: Case report form
CS: Chronic sciatica
GBP: Gabapentin
HLOC: Health Locus of Control Scale
ITT: Intention to treat
NICE-UK: National Institute for Health and Care Excellence – United Kingdom
NP: Neuropathic pain
NSAID: Non-steroidal anti-inflammatory drug
ODI: Oswestry Disability Index
PAGPROS: Pregabalin and Gabapentin Prospective Clinical Trial for the Treatment of Sciatica: A Randomised, Double-Blind, Cross-over Study
PBS: Australian Pharmaceutical Benefits Scheme
PGB: Pregabalin
SAE: Serious adverse event
SE: Side effect
SPIRIT: Standard Protocol Items: Recommendations for Interventional Trials
TCA: Tricyclic antidepressant
VAS: Visual analogue scale
